# Subgroup-based model selection to improve the prediction of vancomycin concentrations

**DOI:** 10.1128/aac.00174-25

**Published:** 2025-07-23

**Authors:** Hanna Kadri Laas, Tuuli Metsvaht, Kadri Tamme, Juri Karjagin, Kristiina Naber, Artjom Afanasjev, Carmen Tiivel, Irja Lutsar, Hiie Soeorg

**Affiliations:** 1 Department of Microbiology, University of Tartu 37546, Tartu, Estonia; 2 Anaesthesiology and Intensive Care Clinic, Tartu University Hospital 37544, Tartu, Estonia; 3 Children’s Clinic, Tartu University Hospital 37544, Tartu, Estonia; 4 Department of Paediatrics, University of Tartu 37546, Tartu, Estonia; 5 Department of Anaesthesiology and Intensive Care, University of Tartu 37546, Tartu, Estonia; Bill & Melinda Gates Medical Research Institute, Cambridge, Massachusetts, USA

**Keywords:** individualized dosing, model-informed precision dosing, genetic algorithm, decision tree, decision algorithm, model selection, external evaluation, critically ill patients, intensive care unit patients

## Abstract

Individualized dosing of vancomycin is recommended, model-informed precision dosing (MIPD) being the preferred method to improve efficacy and limit toxicity. However, its implementation poses challenges, including model selection and initiation dose determination. We developed a model selection tool (MST) and evaluated its potential to improve concentration prediction precision and reduce bias. Retrospective data from adult intensive care unit patients receiving intravenous vancomycin were collected and divided into training and validation data sets. Population predictions from published one-compartment models were computed, and the universally best-performing model (UBM) was selected. A genetic algorithm was used to create an MST. The ability to forecast the third concentration based on previous concentrations was evaluated. A total of 148 vancomycin treatment episodes were included in training and 67 in the validation data set. The MST showed 12% and 6% improved precision compared to the UBM in training and validation data sets, respectively (mean absolute percentage prediction error [mean PAPE] 22.8% vs 26.0% and 28.4% vs 30.2%). The UBM exhibited lower bias in both training and validation data sets (mean percentage prediction error [mean PPE] 5.8% vs 4.7% and −2.8% vs –1.5%, respectively). The MST showed improved performance in predicting the third concentration based on previous concentrations. In both data sets, accuracy was the best/highest when two prior measured concentrations were used (mean PAPE and PPE 17.0% and −3.0% in training and 18.9% and −1.0% in validation data set). Overall, the MST has the potential to enhance vancomycin dosing accuracy from the first dose and simplify model selection, facilitating the utilization of MIPD in clinical practice.

## INTRODUCTION

Serious infections are still a major global health problem, with sepsis-related deaths accounting for almost 20% of global deaths, with a fatality rate of about 40% (1, 2). Sepsis is treatable, but timely implementation of targeted interventions is important, as it improves outcomes (2, 3).

Vancomycin is a drug widely used in the treatment of serious infections caused by Gram-positive bacteria, including methicillin-resistant *Staphylococcus aureus* (MRSA) (4). Its narrow therapeutic window and large interindividual pharmacokinetic (PK) variability warrant the need for therapeutic drug monitoring (TDM) for numerous patient groups, including those hospitalized in the intensive care unit (3, 5–7).

According to the latest guidelines, model-informed precision dosing (MIPD) is referred to as a preferred method of TDM in the treatment of MRSA (3, 7). MIPD improves therapeutic target attainment, decreases the time to achieve the target, and reduces the risk of adverse effects (3, 5, 8, 9). However, there are several problems with implementing such methods in everyday practice, including selecting the best-performing model and determining the initiation dose.

More than 70 different vancomycin PK models have been developed in various populations, and the predicted PK profiles vary markedly with each population PK model (10–39). Therefore, the model used to adjust dosing in a patient needs to be carefully chosen considering factors like disease status, organ function, and body composition (5). As has been admitted before, using a single model that best fits a historical group might not be the best option and is laborious and expensive to find for each group (40–42).

New approaches are needed to streamline this process and enable widespread implementation of MIPD in clinical practice (5, 41, 43). Today, only a few model-selection and model-averaging solutions have been developed, in most cases applying a handpicked selection of models (40). Despite that, they have been found to outperform the commonly used approach of a universally best model, a risk of biased model preselection exists (40).

We aimed to develop a computer-based population PK model selection tool (MST) based on the subgroup identification of patients according to clinical and demographic characteristics for choosing the best-performing model for each patient subgroup.

## MATERIALS AND METHODS

### Selection of data set

For the training data set, hospital records of adult patients having vancomycin concentration measured during the treatment in Tartu University Hospital (TUH) between March 1, 2020 and March 31, 2022 were retrospectively identified from the laboratory database. We included all cases where intravenous treatment with vancomycin was started in TUH and at least one vancomycin blood concentration was measured during third-level intensive care unit (ICU) stay. Vancomycin treatment episodes with a gap of ≥7 days were accounted as separate episodes. For the validation data set, prospectively collected data following the same inclusion criteria between April 1, 2022 and April 31, 2023 were used.

### Data collection and interpretation

Data were collected 2 days prior to the start of the treatment episode and daily during vancomycin treatment. All variables tested as covariates in previously published vancomycin PK models were recorded. In addition, data on survival at the end of ICU and hospital stay were collected. Vancomycin concentrations were measured until February 2021 using Fluorescence Polarization Immunoassay (Roche/Vancomycin) and since then using Kinetic Interaction of Microparticles in Solution (Roche/Vancomycin Gen.3).

Hospital guidelines active during the study period recommended vancomycin dosage of 1,000 mg twice a day in case of creatinine clearance >50 mL/min and 1,000 mg once, with the following doses adjusted according to trough concentration in case of creatinine clearance <50 mL/min (44). Creatinine clearance was calculated using the following formula: {[(140 − *age(yr*)) × (*ideal body weight (kg*))] / (*creatinine in blood (µmol/l*))} × 0.85 (*if female)* (ideal body weight: female = *45 kg +1 kg for every cm of height exceeding 150 cm;* male *= 50 kg +1 kg for every cm of height exceeding 150* cm). Trough concentration (Ctrough) was defined as the concentration measured between 2.5 h and 0.5 h after the scheduled administration of the next dose. Accepting a wider timeframe is based on the structure of the real-world data.

Retrospectively collected data were screened for errors by an independent and experienced auditor in the middle of the data collection phase, when 36.9% (*n* = 59) of the final data set was collected. Five vancomycin treatment episodes were randomly selected (https://www.randomizer.org/), and all transcribed data were screened for errors. Only 1.5% of 9,128 screened data fields were found to be erroneous.

The studies were approved by the Ethics Committee of the University of Tartu (359/T-20, 359/T-13). Informed consent from participants was waived based on national regulatory legislation.

### Formation of MST development and validation data sets

Only the ICU period data of the training data set was used to develop the MST and select the universally best-performing model. Recognizing the dynamic pharmacokinetics in ICU patients, treatment time in the ICU was further divided into two subsets according to the presence of renal replacement therapy. Time in each subset was divided into timeframes so that each timeframe started either with the initiation of treatment or a vancomycin concentration measurement. Each timeframe had to include an additional two measured concentrations. Only the timeframes with a length of ≤72 h were included in further analysis, to avoid too long periods when pharmacokinetics may change considerably. For validation, a similar approach of dividing data into timeframes was applied to the prospectively collected validation data set.

Missing laboratory parameters were imputed with the closest value within ±48 hours. Other missing values were imputed using data from the nearest time points in the following preferential order: 1 day prior, 1 day after, 2 days prior, and 2 days after. If no data from these time points were available, the missing values remained unfilled. All covariates within the timeframes were set to the values of the first timepoint of each timeframe, as these data would be available for real-life forecasting. If the first time point value was missing, it was replaced with the closest in time, or the timeframe would be excluded if no value was present.

### Pharmacokinetic model selection

We included all vancomycin population PK models for adults published from the inception of PubMed until December 22, 2021. PK models published before May 2019 were extracted from two reviews by Marsot et al. and Aljutayli et al. (34, 35). To identify studies that developed population PK models of vancomycin in adults and were published after the inclusion period of the above-mentioned reviews (published from May 1, 2019 to December 22, 2021), a separate PubMed search with the similar search term “vancomycin AND (population-pharmacokinetic* OR nonlinear-mixed-effect OR nonlinear-mixed-effects OR NONMEM) NOT (child*)” was performed on December 22, 2021 (37, 38).

All one-compartment models were included in the MST development process. We excluded two-compartment models as the development of two-compartment models is more complex and more sensitive to model misspecification risk if data are sparse in the development process, while the one-compartment models have been shown to perform comparably well (45, 46). Including more parameters in the model requires more data to avoid overparameterization and overfitting (8).

### Identification of the universally best-performing model

The universally best-performing model in the MST development cohort’s training data set was identified. For all timeframes, population predictions from each included population PK model were calculated. The best-performing model was selected based on the lowest sum of the absolute values of mean percentage prediction error (PPE; PPE = (*measured concentration − predicted concentration*)/*measured concentration* × 100%) and mean percentage absolute prediction error (PAPE; PAPE = *|measured concentration − predicted concentration*|*/measured concentration* × 100%) to account for both, bias and precision.

### Developing a model selection algorithm

Model selection by genetic algorithm was used to find the models’ subset into which patients can be grouped according to their best-performing model, as described by us previously (42). In brief, the genetic algorithm is an optimization algorithm that imitates evolution mechanisms (47). Genetic algorithm iteratively creates numerous models’ subsets, using 10-fold cross validation (CV) to configure a classification and regression tree (CART) and assesses the models’ subsets using a fitness function (mean of mean PAPE of 10 CV sets) to find an optimal subset. The method was chosen as it showed the best results in the preliminary study (42). The method is further described in Text S1.

Assessment of the performance in terms of mean PPE and mean PAPE of the final model selection tool was conducted in the MST development cohorts’ training and validation data set divided into timeframes as described above. First, the ability to select the most suitable model was assessed. The best-performing model for each timeframe was selected according to the lowest PAPE of population predictions. Thereafter, MST was used to suggest the best-performing model. The percentage of overlap between the real best-performing and the MST estimated best-performing model was assessed.

### Assessment of forecasting ability

MST forecasting ability was assessed on the unmodified training and validation data sets, called the assessment cohort. The assessment cohort included treatment episodes that started in the ICU, had ≥3 vancomycin concentration measurements available during the treatment episode, the 3rd concentration was Ctrough, and no covariates needed for prediction were missing. In the evaluation process, the 3rd measured concentration was predicted at the time of the 2nd concentration measurement in the following settings: (1) *a priori* prediction with a universally best-performing model using only the patient’s covariates; (2) *a priori* prediction using developed MST with only the patient’s covariates; (3) patient’s covariates and the 1st plasma vancomycin concentration; (4) patient’s covariates and the 2nd plasma vancomycin concentration; and (5) patient’s covariates and both measured plasma vancomycin concentrations. Mean PPE and mean PAPE were used for assessment of bias and precision.

NONMEM version 7.4 (ICON Development Solutions, MD, USA) was used to calculate population predictions using population pharmacokinetic models. All other data analyses were performed using R (last accessed 25.05.2024).

## RESULTS

### Population

Overall, 651 admissions, with ≥1 available vancomycin concentration, were screened for the training data set (Fig. S1). Among them, 171 had ≥1 vancomycin concentration measured in the ICU, and 160 with 173 treatment episodes were available for inclusion in the study. Prospective data collection for the validation data set yielded 84 admissions, 75 of them with 76 treatment episodes, complied with all the exclusion and inclusion criteria (Fig. S1). Altogether, in the MST development cohort, 546 timeframes were in the training and 260 in the validation data set from 148 and 67 vancomycin treatment episodes, respectively (Fig. S1). For the assessment of forecasting ability, 96 vancomycin dosing episodes were included in training and 38 in the validation data set (Fig. S1).

The demographic and clinical characteristics of patients in training and validation data sets were similar, except for the lower number of patients diagnosed with COVID-19 or needing ECMO in the validation data set compared with the training data set (Table 1).

**TABLE 1:** Characteristic of vancomycin treatment episodes included in the MST development and assessment cohorts’ training and validation data sets^*a*^

	MST development cohort		Assessment cohort	
	Training data set (*n* = 148)	Validation data set (*n* = 67)	Training data set (*n* = 96)	Validation data set (*n* = 38)
General information				
Male (n)	113 (76.4%)	43 (64.2%)	77 (80.2%)	25 (65.8%)
Age (years)	63.0 (55.0–70.0)	67.0 (53.0–73.0)	62.5 (52.8–70.3)^*c*^	70.0 (61.5–73.0)^*c*^
Weight at the beginning of VAN treatment (kg)	81.8 (70.0–95.0)	80.5 (63.8–99.0)	84.5 (72.9–101.1)	84.5 (63.8–99.0)
BMI at the beginning of VAN treatment (kg/m^2^)	27.4 (24.0–31.1)	26.5 (21.7–34.5)	28.8 (24.2–32.0)	27.3 (22.7–35.4)
Usage of inotropes (n)^*b*^	124 (83.8%)	58 (86.6%)	79 (82.3%)	32 (84.2%)
Invasive ventilation (n)^*b*^	107 (72.3%)	49 (73.1%)	74 (77.1%)	28 (73.7%)
ECMO during VAN treatment (n)^*b*^	16 (10.8%)^*c*^	1 (1.5%)^*c*^	13 (13.5%)^*c*^	0 (0.0%)^*c*^
Isolated Gram+ bacteria (n)^*d*^	56 (37.8%)	25 (37.3%)	38 (40.0%)	17 (44.7%)
RRT during VAN treatment (n)^*b*^	47 (31.8%)	15 (22.4%)	28 (29.2%)	9 (23.7%)
APACHE II score (n)^*e*^	23.0 (18.0–30.0)	24.0 (18.0–30.0)	22.0 (17.0–29.0)	23.5 (17.0–30.0)
SOFA score (n)^*e*^	6.0 (4.0–9.0)	6.0 (5.0–8.0)	6.0 (4.0–8.3)	6.5 (5.0–9.0)
COVID-19 (n)^*b*^	61 (41.2%)^*c*^	14 (20.9%)^*c*^	40.0 (41.7%)^*c*^	5 (13.2%)^*c*^
VAN treatment characteristics				
VAN treatment duration (days)	8.0 (5.0–13.0)	10.0 (6.0–14.0)	7.5 (5.0–12.0)	9.5 (6.3–12.0)
Doses per treatment episode in ICU (n)	12.0 (8.0–18.0)	13.0 (6.0–20.5)	12 (8.0–18.0)	14.5 (9.3–20.8)
Concn per treatment episode in ICU (n)	7.0 (5.0–11.0)	8.0 (4.0–13.0)	7.5 (5.0–12.0)	9.0 (4.3–13.0)
Ctrough per treatment episode in ICU (n)	5.0 (2.0–8.3)	5.0 (3.0–10.0)	6.0 (4.0–10.0)	7.0 (3.3–11.0)
Ctrough (n)	1155 (69.1%)	561 (68.8%)	839 (81.1%)	341 (73.5%)
Mean Ctrough for each treatment episode (mg/L)	17.2 (14.3–19.0)	17.1 (15.1–19.5)	16.9 (4–18.8)	17.0 (15.3–19.5)
Mean creatinine for each treatment episode (µmol/L)	84.7 (57.2–130.1)	88.2 (64.0–131.4)	77.0 (59.3–118.8)	78.6 (64.3–129.4)
Mean eGFR for each treatment episode (µmol/L)	83.9 (54.8–106.3)	80.6 (54.1–98.6)	87.7 (63–107.9)	84.9 (53.9–98.6)
Characteristics of timeframes				
Total number of timeframes (n)	546	260	N/A	N/A
Number of timeframes per VAN treatment episode (n)	3 (2.0–4.3)	3 (1.0–6.0)	N/A	N/A
Timeframe duration (h)	45.6 (24.4–48.0)	38.1 (24.3–48.0)	N/A	N/A
Characteristics of VAN concentrations and VAN concentration intervals				
Interval between 1st and 2nd concn (h)	N/A	N/A	22.9 (12.5–24.0)	17.2 (10.1–23.9)
Interval between 2nd and 3rd concn (h)	N/A	N/A	23.3 (12.1–24.0)	23.6 (13.0–24.2)
Time of 1st concn (h)	N/A	N/A	22.4 (21.1–23.7)	23.2 (21.9–34.6)
Time of 2nd concn (h)	N/A	N/A	45.9 (34.9–47.3)	45.8 (35.0–47.1)
Time of 3rd concn (h)	N/A	N/A	69.6 (46.8–70.9)	70.0 (48.5–70.7)

^*a*^ Data are presented as median (interquartile range of 1st and 3rd quartiles) or total counts (%).

^*b*^ Counted positive if present at least 1 day during VAN treatment.

^*c*^ Significant *P* value.

^*d*^ In case 2, days before the beginning or during VAN treatment taken analysis from blood or sterile body fluids that included any Gram+ bacteria, the colonization with Gram+ bacteria was counted as positive.

^*e*^ APACHE and SOFA scores calculated according to data from the 1st 24 hours in ICU APACHE, acute physiology, and chronic health evaluation; BMI, body mass index; concn, concentration; Ctrough, trough concentration; ECMO, extracorporeal membrane oxygenation; ICU, intensive care unit; N/A, not available; RRT, renal replacement therapy; SOFA, sequential organ failure assessment; VAN, vancomycin.

### Model selection

**Fig 1:**
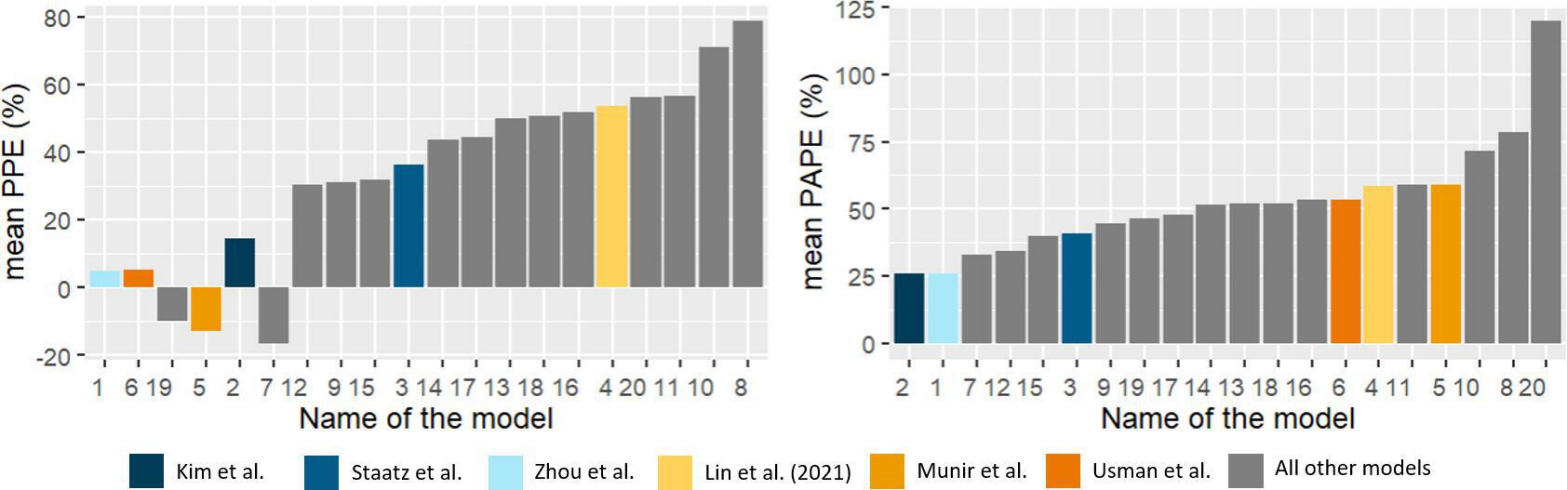
The selection of a universally best-performing model (ZHOU by Zhou et al., marked with light blue) based on the lowest mean PPE and mean PAPE. The six models in the final subset of models identified by the genetic algorithm and used for the final classification regression tree are colored; all other models are gray. Mean PPE, mean percentage prediction error; mean PAPE, mean absolute percentage prediction error. All the numbers represent models as follows: 1, Zhou et al.; 2, Kim et al. (all patients); 3, Staatz et al.; 4, Lin et al. (2021); 5, Munir et al.; 6, Usman et al.; 7, Medellin-Garibay et al.; 8, Masich et al.; 9, Deng et al.; 10, Alqahtani et al. (carcinoma); 11, Alqahtani et al. (no carcinoma); 12, Kim et al. (neurosurgical patients); 13, Buelga et al.; 14, Adane et al.; 15, Ji et al.; 16, Jing et al. (2019); 17, Kovacevic et al.; 18, Lin et al. (2016); 19, Udy et al.; 20, Wu et al.

Altogether 20 models were included in analysis (Fig. S2), with mean PAPE ranging from 25.8% to 119.8% and absolute value of mean PPE ranging from 4.7% to 78.7% (Fig. 1). The universally best-performing model according to the bias and precision was the one by Zhou et al., as it had the lowest sum of absolute values of mean PPE and PAPE (30.7%), with lowest mean PPE (4.7%) and the second lowest mean PAPE values (26.0%) (Fig. 1).

### Final MST

**Fig 2:**
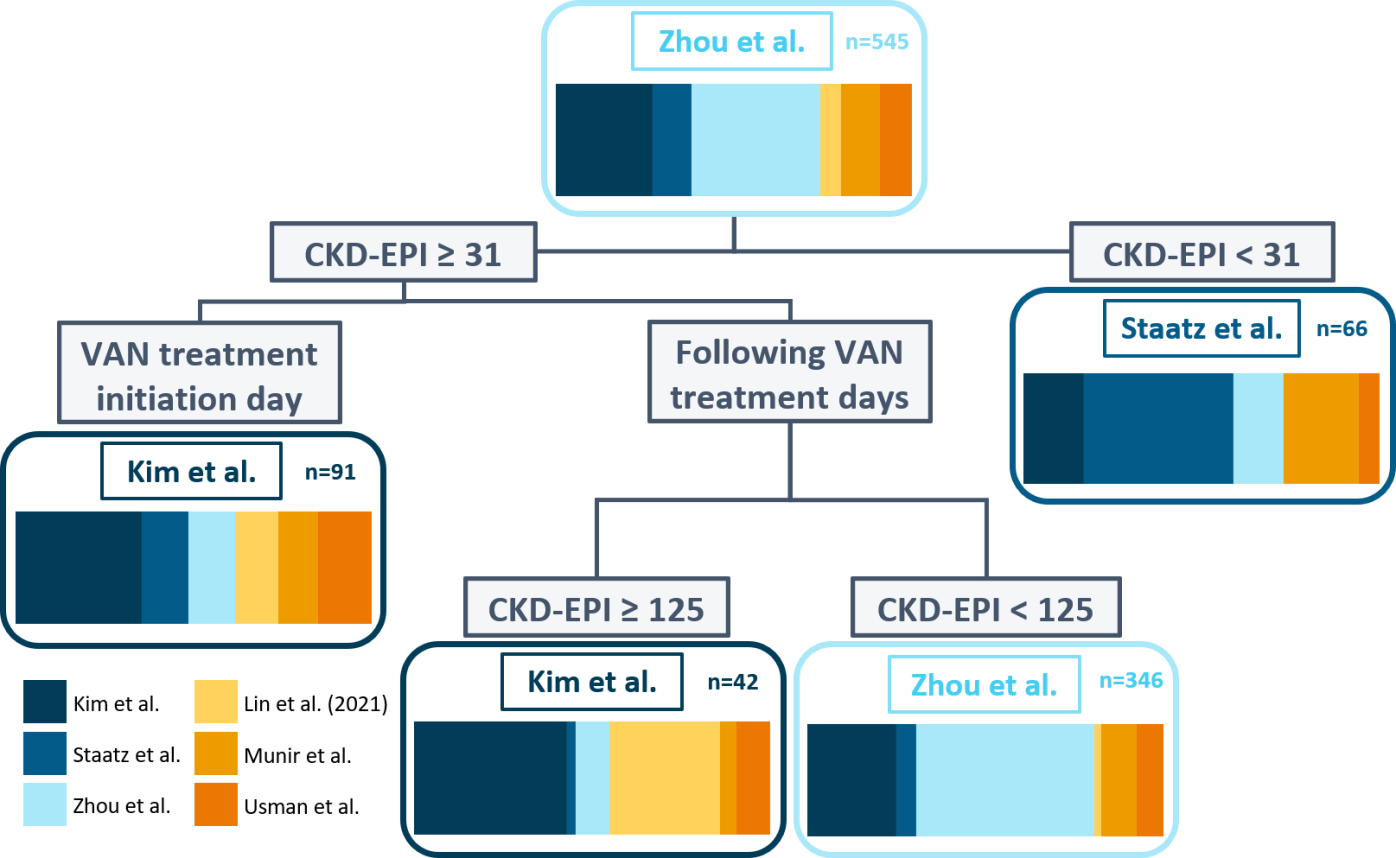
Final MST with a depth of 3 was developed using a group of 6 models (color-coded). Final MST divides patients between three different previously published pharmacokinetic models according to the day of treatment and glomerular filtration rate assessed by the CKD-EPI equation (mL/min/1.73 m²). The colored bar chart shows the proportion of the best-performing models for data frames in that division. CKD-EPI, Chronic Kidney Disease Epidemiology Collaboration; VAN, vancomycin.

Using a genetic algorithm and data from the MST development cohorts’ training data set, a final subgroup of six models (Kim et al. [36]; Staatz et al. [37]; Zhou et al. [38]; Lin et al. [19]; Usman et al. [39]; Munir et al. [16]) was identified for the MST (Table S2). The selected subgroup had the lowest mean of the mean PAPEs of 10 CV sets in the final generation of the genetic algorithm (22.8%; Table S3). The final MST uses kidney function assessed by the CKD-EPI formula and vancomycin treatment day to select the most suitable of the three models (Kim et al. [36]; Staatz et al. [37]; Zhou et al. [38]) (Fig. 2). The final MST assigned the individually best-performing model, from the six model subgroups, to 62.4% in the training and 46.5% of the patients in the validation data set of the MST development cohort.

The final MST showed an improvement in precision of 3.2 and 1.8 percentage points compared to the universally best-performing model in the training (mean PAPE 22.8% vs 26.0%) and validation data sets (mean PAPE 28.4% vs 30.2%) of the MST development cohort. The universally best-performing model exhibited lower bias in training (mean PPE 4.7% vs 5.8%) and in the validation data set (mean PPE −1.5% vs −2.8%).

### Assessment of forecasting ability

**Fig 3:**
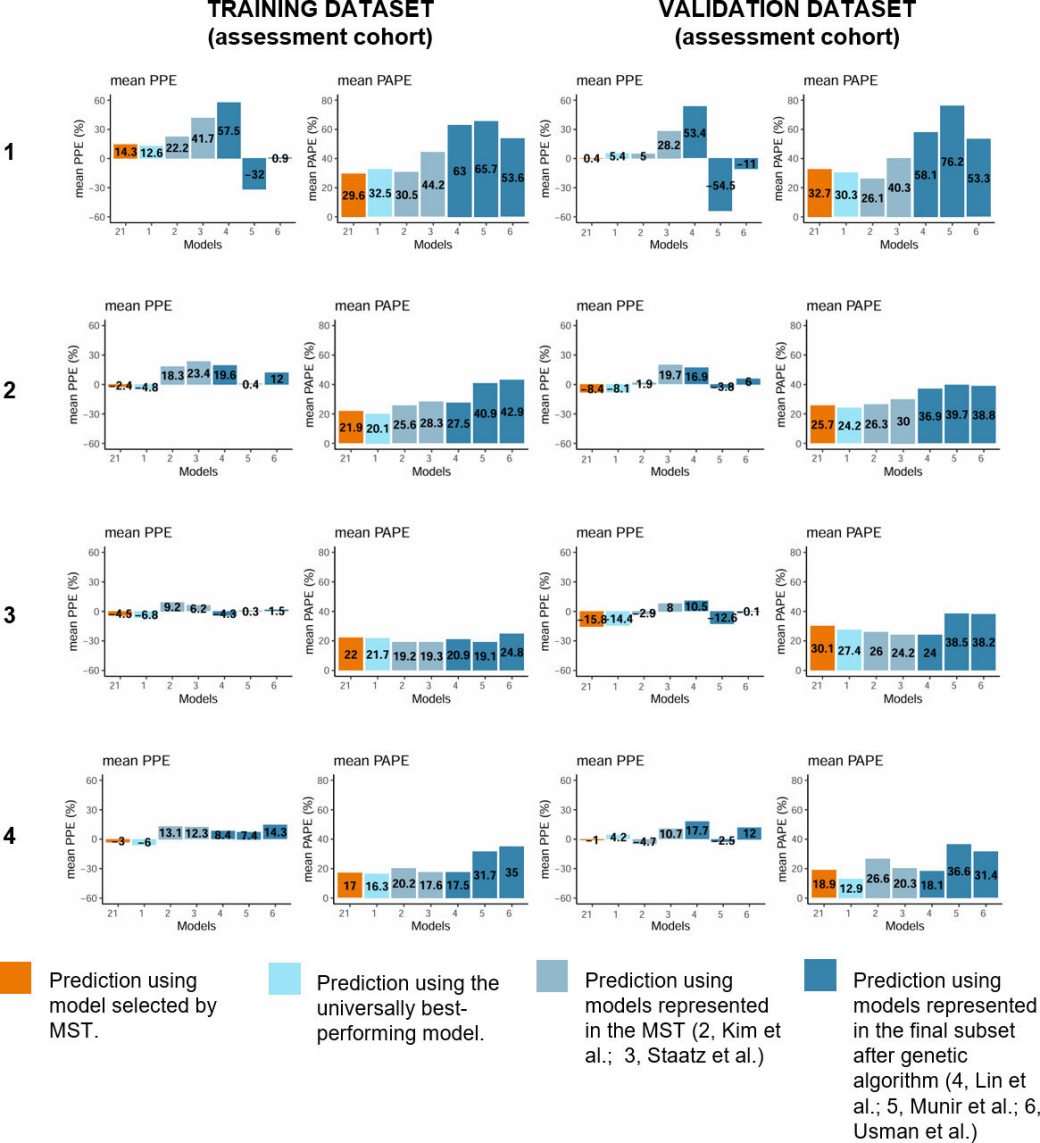
Predicting concentration-time data of the third measured concentration, which is blinded to the model/algorithm, in the assessment cohorts training and validation data sets in various settings: (1) a priori prediction using only the patient covariates; (2) prediction using patients covariates and the first plasma vancomycin concentrations; (3) prediction using patients covariates and the second plasma vancomycin concentrations; and (4) prediction using patients' covariates and both plasma vancomycin concentrations. All the numbers represent models as follows: 1, Zhou et al.; 2, Kim et al. (all patients); 3, Staatz et al.; 4, Lin et al. (2021); 5, Munir et al.; 6, Usman et al.; 21, Prediction using model selected by MST.

The a priori prediction using the final MST resulted in lower precision compared to the prediction of the universally best-performing model in the validation data set, but the bias was higher for the universally best-performing model (Fig. 3, Table S4). Using the 1st concentration resulted in improved precision and bias compared to a priori predictions in most cases (Fig. 3, Fig. S3). If only the closest concentration to the predicted concentration (the 2nd concentration) was used, the bias and precision showed improvement in many cases in the training set. Less noticeable improvement was seen in the validation data set. For the universally best-performing model and final MST, the bias and precision were both worse compared to using the 1st concentration in training and in validation data sets (Fig. 3).

The bias and prediction error of the MST, as well as the universally best-performing model, were lowest using two prior measured concentrations in training (MST: mean PPE −3.0%, mean PAPE 17.0%; best-performing model: mean PPE −6%, mean PAPE 16.3%; Fig. 3) and validation (MST: mean PPE −1.0%, mean PAPE 18.9%; best-performing model: mean PPE 4.2%, mean PAPE 12.9%; Fig. 3) data sets.

The performance of the model by Kim et al., suggested by MST for dose calculations on the first day, was comparable to MST and the model by Zhou et al., showing one of the highest precisions and lowest biases for a priori predictions in training and the highest precision in the validation data set (Fig. 3, Fig. S3).

Both in training and validation data sets, the best-performing model selected for the assessment of forecasting ability by MST was mostly Zhou et al. (72 and 30 times out of 96 and 38, respectively). Staatz et al.’s model was the second most suggested model in the validation data set (Staatz et al. six times; Kim et al. two times); in the training data set, Staatz et al. and Kim et al. were both suggested 12 times.

## DISCUSSION

In this study, we developed a model selection tool for vancomycin dose optimization in adult ICU patients. The developed MST uses available patients’ data, groups them into smaller cohorts, and then suggests the best-fitting PK model is suggested. No single model resulted in overall better performance than MST. The good performance of MST is confirmed by equally good performance in the validation data set. However, the changes between the MST and best-performing model for the overall population (Zhou et al.) were rather small, being below 20% [10]. While changes below 20% may be considered clinically insignificant, our comparison was made against the best-performing model for our population—especially relevant given that the optimal model remains unknown in many populations. Notably, the improvement is more substantial when compared to several other models (Fig. S3). Therefore, the developed tool is still a good alternative to using a subjectively chosen single model in settings where the best-performing model has not been established. The dosing tool will be available at website https://dosesage.app/.

This study follows a previously published proof-of-concept study conducted with the data of neonates receiving vancomycin (42). Genetic algorithm for the development of the MST was chosen as the best-performing approach (42). There are other available solutions to tackle the complexity of the model selection process. A less complex solution would be selecting the model for each subgroup according to the population it was developed (48). However, such an approach does not consider the potential heterogeneity within groups. Furthermore, it may be complicated when a patient meets inclusion criteria for multiple subgroups that have separate models (e.g., obese neurosurgical patients). A well-working solution of combining models to improve precision has been proposed by Uster et al., but they included only a handful of preselected models in development (40). It also cannot be used for *a priori* dose prediction, the most challenging as well as important part, as early appropriate antibiotic exposure has been associated with better treatment results (49). In our study, data were used to determine the best selection from one-compartment models via the genetic algorithm. As has been shown previously and can also be seen in this study, a population-wide poorly performing model, like Staatz et al., may prove the most suitable for some patients (37, 42).

Information use was further maximized by the inclusion of a large number of covariates. We used CART analysis to decide the final selection as well as the most suitable cut-off points. The final decision tree structure is well supported by existing knowledge about vancomycin PK. Unsurprisingly, the final MST included kidney function, the most important factor affecting vancomycin PK (50). First division of MST sorts out people with very low glomerular filtration rate (CKD-EPI <31 mL/min/1.73 m^2^) and recommends using the model by Staatz et al. (37). Compared to other models included in the final MST, Staatz et al.’s development population had relatively high serum creatinine values that might make this model more suitable to patients with lower renal function (creatinine values: median 101 µmol l^−1^ in Staatz, mean of 90.6 ± 31 µmol/L in Zhou and 76.93 ± 40.67 µmol/L and 56.6 ± 21.2 µmol/L in Kim et al. [non-neurosurgical and neurosurgical patients, respectively]) (36–38).

There might be various causes why treatment day has emerged as an important subdivision. Some models may perform better *a priori* or at the beginning of treatment than others. The model by Kim et al. is recommended by MST for patients with CKD-EPI ≥31 mL/min/1.73 m^2^ on their first treatment day. In our study, this model was the best performing for *a priori* predictions, as it had the lowest mean PAPE and mean PPE in the validation set and better accuracy for *a priori* prediction in the training set as well. Interestingly, additional TDM data did not improve this model’s ability to perform. The reason why the model by Kim et al. is suggested for the predictions on the first day may be that it includes early (≤72 h from the treatment initiation) and late treatment as a covariate (36). They argued that before achieving distribution equilibrium, the drug concentration decreases faster than the amount of drug in the body, resulting in an increase in volume of distribution over time (36). The model by Kim et al. is also recommended in case of very high glomerular filtration rate (CKD-EPI ≥125 mL/min/1.73 m^2^) (36). The development set for this model included patients with rather low serum creatinine levels (36).

For dose recommendations on later treatment days and with regular estimated glomerular filtration rate (CKD-EPI 31-125 mL/min/1.73 m^2^), MST recommends using the model by Zhou et al., the universally best performing model in our data set.

We acknowledge that the novel guidelines recommend AUC/MIC-based targets for therapeutic drug monitoring when calculating the suitable dose for vancomycin. Also, the developed model selection and dosing tool allows choosing a suitable target (AUC/MIC, concentration, or combination of AUC/MIC and concentration) for dose predictions. However, in the MST developing process, we used the concentration to evaluate the predictive performance of models in our population. In contrast to AUC, which is a calculated value, concentration is actually measured. Estimating a valid reference AUC (“true AUC”) based on mostly Ctrough data poses some problems. Ideally, to create adequate AUC reference data, a rich-sampling PK study would be needed, which is not feasible in a large population like ours. Alternatively, to calculate AUC, we need a good-performing model. The current study aimed to find the best model(s) for the population.

In some other studies, AUCs have been calculated based on a model. In a previous study by Uster et al., a single model developed by Goti et al., with re-estimated model parameters, was used for calculating reference AUC, although no data were provided about the actual fit on AUC data (1). However, by the approach of re-estimating model parameters, they also attempted to first get the best-fitting model to the population.

It has also been acknowledged in other studies that reliable predictions of AUC are difficult to obtain, but assessment of predictive performance provides support for suitability for AUC prediction (2). Numerous studies reported good correlation between AUC and Ctrough (3–7). Therefore, due to the uncertainty about AUC estimation, we considered relying on measured concentrations in the development of our model selection tool (assessment of prediction accuracy and bias) to be a sounder approach.

Our MST’s advantage is the possibility to be used to tailor the dosing scheme from the first dose, with smaller bias for *a priori* predictions compared to other models. The MST, compared to a universally best-performing model, offers more stability across a wide range of patients in prediction accuracy in *a priori* as well as later predictions. In line with previous studies applying the single model approach, the prediction precision and bias improve when more individual concentrations are available (10, 48). Interestingly, some previous model selection approaches have shown no or minimal improvement with using additional concentration data (40).

### Limitations

Our study has some limitations. First, due to using real-world data, there is a reasonable opportunity for some imprecision in the data of the hospital records. To ensure that data insertion quality does not limit the research quality, we performed a screening of retrospectively reviewed hospitalization records. The actual error rate was comparable to or lower (1.5%) than confirmed in similar studies and much lower than the estimated acceptable (4.93%) (51, 52). Still, the use of data from a single center may impact the MST’s applicability in different settings.

Due to using real-world data, a wider timeframe was accepted for the Ctrough. Dose administration times may be inaccurately documented in clinical practice with infusions started within up to 1 hour after the scheduled time, while TUH routine practice excludes measuring peak concentrations or concentrations during infusion. Therefore, and to avoid exclusion of any real-life scenarios with potential impact on our predictions, concentrations 2.5 h before to 0.5 h after the scheduled start of infusion were treated as Ctrough in this study. All the concentrations collected 0–0.5 h after the scheduled start of vancomycin infusion were manually checked for unusual values, but none were found. Dose administration times may be inaccurately documented in clinical practice, but the effect on the predictions has shown a minimal effect for vancomycin. Half-hour deviation from marked time has been shown to result in a 0.1–3.9% increase in cases that are incorrectly estimated to reach the area under the concentration-time curve during a 24 hour period (AUC_24_)/minimum inhibitory concentration (MIC) >400 (53).

Second, our training and validation populations differed by the use of ECMO and by the number of patients being diagnosed with COVID-19 during the hospitalization. We estimate that they are connected, as the training data set was collected during the most severe COVID-19 pandemic time (2020–2021) (54). However, we believe that these differences have little, if any, impact on the result, as previous studies and our analysis (unpublished data) have shown no significant difference in vancomycin PK during ECMO treatment (55). This is confirmed by the good performance of our MST in the validation data set.

Third, there was also a difference between patients’ age in the training and validation sets of the assessment cohort. Age has been tested as a covariate for most of the models; however, it remained a significant covariate in only a few. For example, in the reviewed 29 models, 28 tested age as a covariate, but only 8 reported it as significant (35). A study conducted among >80-year-old people found only slight differences between the PK of vancomycin compared to younger patients (56). Our developed MST’s acceptable accuracy in slightly different populations confirms its good performance. A clinical trial is ongoing to prove its value in a real working environment.

Fourthly, only one-compartment models were included in the MST development. Most vancomycin population models are developed from retrospective TDM data, which focus on trough samples (46). Two-compartment models developed with trough-only data sets have been shown to perform worse compared to two-compartment models developed with peak-trough data sets (46, 57). One-compartment models developed with sparse data, on the other hand, have been shown to perform comparably well with two-compartment models developed with peak-trough data (45, 46, 58). As one-compartment models are more robust to errors made in the development process, are simpler, and are expected to perform comparably well, we preferred to include only one-compartment models.

Fifth, we acknowledge that the differences between the MST and the best-performing model in all tested settings are relatively small and, possibly with limited clinical benefit, especially given the added complexity. To truly justify the use of MST, a further analysis on the impact of actual dosing and the probability of over-/under-dosing of future patients would be ideal, but remains beyond the scope of this manuscript.

### Conclusions

A developed and evaluated model selection tool for vancomycin dose optimization in adult ICU patients simplifies the model selection process and has good potential to improve the performance and applicability of MIPD in heterogeneous patient cohorts.

## SUPPLEMENTAL MATERIAL

Fig. S1 aac.00174-25-s0001.tif Process of data collection for validation and training datasets.

Fig. S2 aac.00174-25-s0002.tif Selecting models and developing process of the model selection tool.

Fig. S3 aac.00174-25-s0003.tif Predicting concentration-time data of the third measured concentration, which is blinded to the model/algorithm, in the assessment cohorts training and validation datasets in various settings.

Supplemental legends aac.00174-25-s0004.docx Legends for Fig. S1 to S3.

Supplemental text aac.00174-25-s0005.docx Supplemental text for the article.

Supplemental tables aac.00174-25-s0006.docx Tables S1 to S4.

ASM does not own the copyrights to Supplemental Material that may be linked to, or accessed through, an article. The authors have granted ASM a non-exclusive, world-wide license to publish the Supplemental Material files. Please contact the corresponding author directly for reuse.
