# Supplementary figures and images for: Subgroup-based model selection to improve the prediction of vancomycin concentrations
Source: Antimicrob Agents Chemother. 2025 Jul 23;69(9):e00174-25. doi: 10.1128/aac.00174-25 (PMC12406661; doi:10.1128/aac.00174-25)

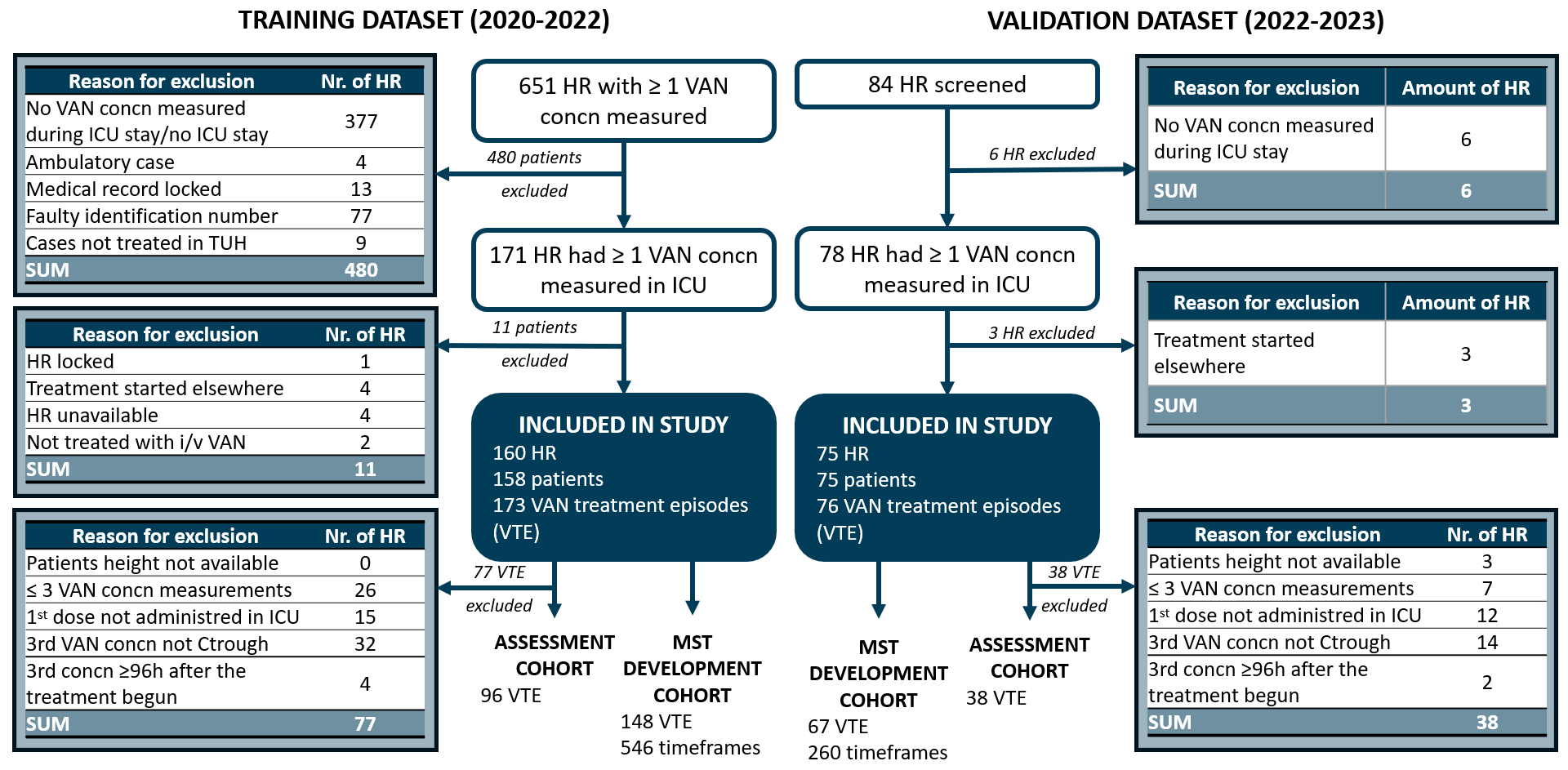

Supplement: Fig. S1 — Process of data collection for validation and training datasets. [file aac.00174-25-s0001.tif]

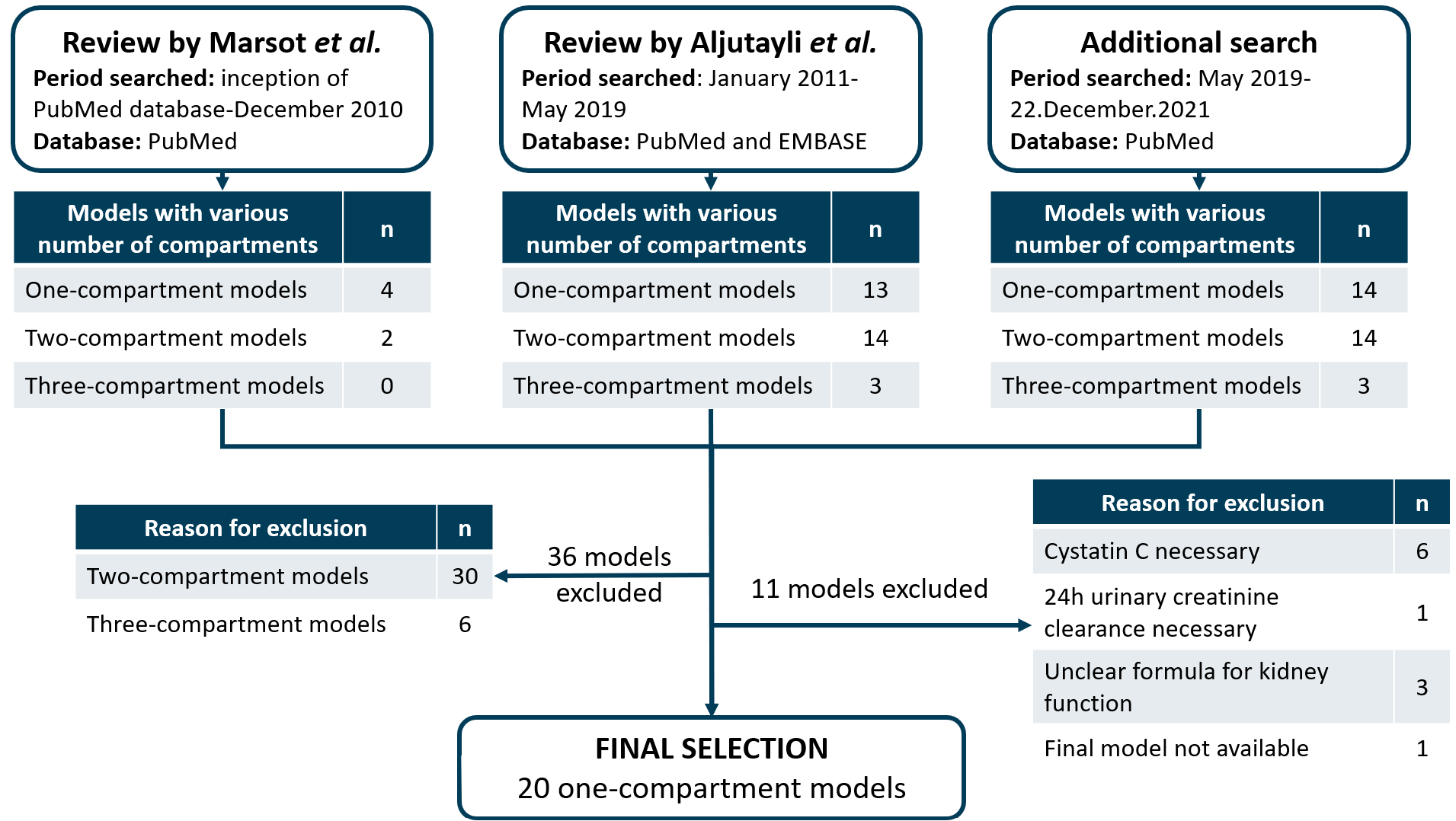

Supplement: Fig. S2 — Selecting models and developing process of the model selection tool. [file aac.00174-25-s0002.tif]

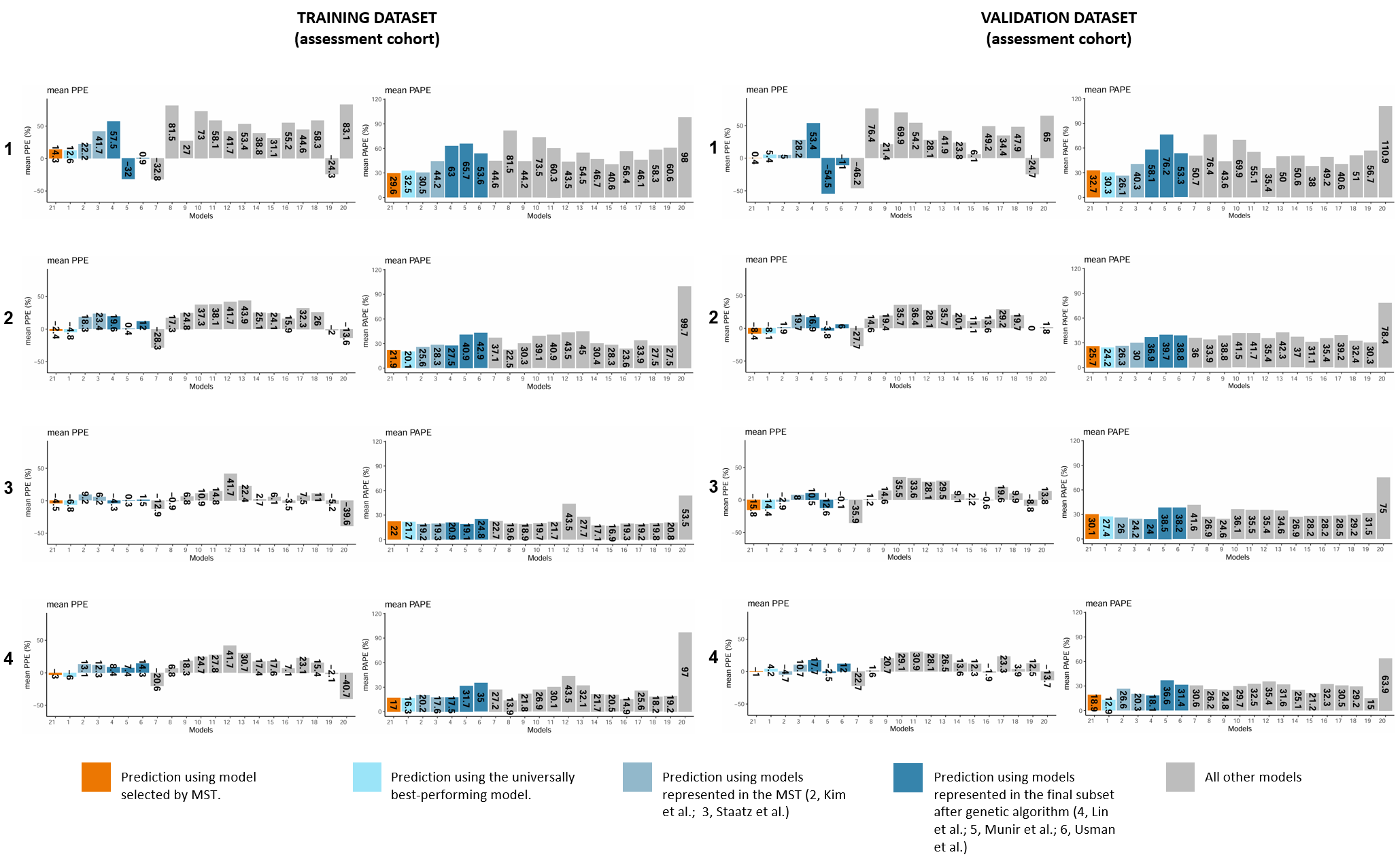

Supplement: Fig. S3 — Predicting concentration-time data of the third measured concentration, which is blinded to the model/algorithm, in the assessment cohorts training and validation datasets in various settings. [file aac.00174-25-s0003.tif]
