# Supplementary material for: Subgroup-based model selection to improve the prediction of vancomycin concentrations
Source: Antimicrob Agents Chemother. 2025 Jul 23;69(9):e00174-25. doi: 10.1128/aac.00174-25 (PMC12406661; doi:10.1128/aac.00174-25)
Supplement: Supplemental legends — Legends for Fig. S1 to S3. [file aac.00174-25-s0004.docx]

**Supplementary figure legends for manuscript “Subgroup based model selection to improve prediction of vancomycin concentrations”**

**Figure S1.** Process of data collection for validation and training datasets. Both datasets were used in analyses with timeframes including 148 and 67 VTE respectively. In validation analyses 96 and 38 VTE were used. Ctrough, trough concentration; concn, concentration; HR, hospitalisation record; ICU, intensive care unit; TUH, Tartu University Hospital; VAN, vancomycin; VTE, vancomycin treatment episode.

**Figure S2.** Selecting models and developing process of the model selection tool. PK models published before May 2019 were extracted from two reviews by Marsot et al. and Aljutayli et al.^39,40^. A PubMed search of studies in adults using search term “vancomycin AND (population-pharmacokinetic* OR nonlinear-mixed-effect OR nonlinear-mixed-effects OR NONMEM) NOT (child*)” was performed on December 22, 2021 to identify studies that have developed population PK models of vancomycin in adults published from May 1, 2019 to December 22, 2021. Two- and three-compartment models were excluded from the MST development process. Due to missing necessary covariates in our dataset, unclear covariates and unavailable final models we excluded 11 models. Final selection of 20 one-compartment models were used in final MST development.

**Figure S3.** Predicting concentration-time data of the third measured concentration, which is blinded to the model/algorithm, in the assessment cohorts training and validation datasets in various settings: (1) a priori prediction using only the patient covariates; (2) prediction using patients covariates and the first plasma vancomycin concentrations; (3) prediction using patients covariates and the second plasma vancomycin concentrations and (4) prediction using patients covariates and both plasma vancomycin concentrations. All the numbers represent models as follows: 1, Zhou et al.; 2, Kim et al. (all patients); 3, Staatz et al.; 4, Lin et al. (2021); 5, Munir et al.; 6, Usman et al.; 7, Medellin-Garibay et al.; 8, Masich et al.; 9, Deng et al.; 10, Alqahtani et al. (carcinoma); 11, Alqahtani et al. (no carcinoma); 12, Kim et al. (neurosurgical patients); 13, Buelga et al.; 14, Adane et al.; 15, Ji et al.; 16, Jing et al. (2019); 17, Kovacevic et al.; 18, Lin et al. (2016); 19, Udy et al.; 20, Wu et al; 21, prediction using model selected by MST.
