# Supplementary material for: Subgroup-based model selection to improve the prediction of vancomycin concentrations
Source: Antimicrob Agents Chemother. 2025 Jul 23;69(9):e00174-25. doi: 10.1128/aac.00174-25 (PMC12406661; doi:10.1128/aac.00174-25)
Supplement: Supplemental text — for the article. [file aac.00174-25-s0005.docx]

**Supplementary text “Subgroup based model selection to improve prediction of vancomycin concentrations”**

**Text S1.**

The algorithm started with the formation of random subsets containing M=4,5,6,7,8 models. The largest number of models was restricted to decrease the possibility of overfitting. With each number of models, 100 subsets were arranged. Subsequently, the 5-step process was performed separately within each of the five subsets of M models and repeated in each set 400 times (generations). The genetic algorithm was repeated thrice for each M to ensure the convergence to the most optimal set.

As a first step, fitness was calculated for each subset. For that, the best model from the subset was selected for each timeframe based on mean absolute prediction error (PAPE). After that, using 10-fold cross-validation (CV), a classification and regression tree (CART) with a maximum depth of 4 using 76 different clinical and demographic covariates, as listed in Table S3, was built to predict the best model for each timeframe. Maximum depth was set to 4, as creating a tree with a depth less than four will not allow using all the up to 8 available models, but more immense depths increase the risk of overfitting. The mean mean PAPE of 10 CV sets was used as the fitness metric. For model subsets where at least one model was best performing for <7 patients, the fitness metric was set to 9999 to reduce the inaccuracy of such CART resulting from imbalanced data (some models being the best for very small number of patients, some models for large number). Second, tournament selection was performed, assigning each of the 100 models’ subsets a random opponent from all the remaining 99 subsets. The subset with better fitness was preserved.

The third step is a crossover, where all the subsets were divided randomly into 50 pairs and randomly selected models from those subsets were exchanged. Each subset was included only once, and the maximum number of traded models was M-1. Fourth, in a mutation step, a random replacement of models within each models’ subset was performed with a probability of 1/M. Probability depends on the number of models in the subset to allow an average of 1 model change per subset, as more extensive changes may result in unreasonable calculations and overall worse outcomes. Fifth, in the elitism step, a random models’ subset was replaced with the models’ subset with the highest fitness in the first step to ensure that this is carried forward to the next generation.

After finishing these five steps, each time, the modified set of the initial 100 subsets was carried on to the next generation, where the described five steps were repeated for 400 generations. Fitness was calculated for the final models’ subsets from the last generation.

The final MST was developed using the classification regression analysis with the models’ subset having the lowest mean mean PAPE of the 10 CV sets and 76 different clinical and demographic covariates. CV was not used for the final CART development to include all the training data. The final CART is used in the final model selection tool**.**
