# Supplementary material for: Subgroup-based model selection to improve the prediction of vancomycin concentrations
Source: Antimicrob Agents Chemother. 2025 Jul 23;69(9):e00174-25. doi: 10.1128/aac.00174-25 (PMC12406661; doi:10.1128/aac.00174-25)
Supplement: Supplemental tables — Tables S1 to S4. [file aac.00174-25-s0006.docx]

**Supplementary tables for manuscript “Subgroup based model selection to improve prediction of vancomycin concentrations”**

**Table S1.** Various clinical and demographic covariates used to develop final MST.

|  | **Data item** | **Description of collected data** | **Frequency of data collection** |
| --- | --- | --- | --- |
| **DEMOGRAPHICS** | Sex | Female or male | Collected once during the study period |
|  | Age | Value, unit |  |
|  | Height |  |  |
|  | Bodyweight | Value, unit | Once for each day of treatment with vancomycin and 2 days prior |
|  | Body mass index in the beginning of treatment with vancomycin |  |  |
|  | Body mass index in the beginning of treatment in ICU |  |  |
|  | Lean body weight (calculated using the Janmahasatian formula) |  |  |
|  | Ideal body weight (calculated using Devine formula) |  |  |
|  | Adjusted body weight (AdjBW[kg] = IBW[kg] + 0.4 · (BW[kg] − IBW[kg]); calculated with DiPiro and Devine ideal body weights according to formula) |  |  |
|  | Day of treatment with vancomycin | Value |  |
|  | Neurosurgery (defined according to Nomesco Classification of Surgical Procedures 2011 as any procedure code that begins with AA) | Present/Not-present | Collected once after the end of hospitalisation |
| **FOREIGNCODIES** | Use of mechanical ventilation | Present/Not-present | Collected once during the study period |
|  | Use of ECMO |  |  |
|  | Renal replacement therapy status |  |  |
|  | Presence of central venous catheter |  |  |
| **PHYSIOLOGICAL PARAMETERS** | Minimum body temperature | Value, unit | Once for each day of treatment with vancomycin and 2 days prior |
|  | Maximum body temperature |  |  |
|  | Minimum mean arterial pressure |  |  |
|  | Maximum arterial pressure |  |  |
|  | Minimum heart rate |  |  |
|  | Maximum heart rate |  |  |
|  | Minimum respiratory rate |  |  |
|  | Maximum respiratory rate |  |  |
|  | Diuresis |  |  |
|  | Urine output |  |  |
|  | Total fluid input |  |  |
|  | Total fluid output |  |  |
|  | Total fluid balance |  |  |
|  | Fluid balance accounting for insensible fluid loss |  |  |
| **DRUGS** | Co-administration of nephrotoxic drugs (drugs included: aminoglycosides (amikacin and tobramycin), liposomal amphotericin B, any antiviral agents, colistimethate, cytotoxic agents (such as cytosine arabinoside, fludarabine and idarubicin), cyclosporine, tacrolimus, any non-steroidal anti-inflammatory agents and trimethoprim/sulfamethoxazole, colistin) | Administered/Not-administered | Once for each day of treatment with vancomycin and 2 days prior |
|  | Cyclosporine |  |  |
|  | Tacrolimus |  |  |
| **DRUGS** | Immunosuppressive drugs (drugs included: cyclosporine, tacrolimus, mycophenolate mofetil) | Administered/Not-administered | Once for each day of treatment with vancomycin and 2 days prior |
|  | Glycocorticosteroids set 1 (drugs included: hydrocortisone i.v./p.o. or fludrocortisone p.o.) |  |  |
|  | Glycocorticosteroids set 2 (drugs included: ethylprednisolone i.v./p.o., dexamethasone i.v. or prednisolone i.v./p.o. |  |  |
|  | Use of nonsteroidal anti-inflammatory drugs (drugs included: ibuprofen, dexketoprofen, ketoprofen, ketoral, naproxen) |  |  |
|  | Use of diuretics (drugs included: furosemide, torasemide, spironolactone, indapamide, hydrochlorothiazide, acetazolamide, mannitol, eplerenone) |  |  |
|  | Use of inotropic and vasoactive agents (drugs included: dopamine, dobutamine, noradrenaline, adrenaline, vasopressin, milrinone) |  |  |
|  | Maximum i.v. noradrenaline dose | Maximum administration speed (mg/h) |  |
|  | Use of i.v. furosemide | Cumulative daily dose, unit of the dose, route of administration (i/v or p/o)  Administered/Not-administered |  |
|  | Use of p.o. indapamide |  |  |
|  | Angiotensin converting enzyme inhibitors |  |  |
|  | Amlodipine |  |  |
| **DRUGS** | Any antifungal drug | Administered/Not-administered | Once for each day of treatment with vancomycin and 2 days prior |
|  | Any antiviral drug |  |  |
|  | Filgrastime |  |  |
|  | Heparin |  |  |
| **NUTRITION** | Parenteral nutrition | Present/Not-present | Once for each day of treatment with vancomycin and 2 days prior |
|  | Enteral nutrition |  |  |
| **FLUIDTHERAPY** | Administration of balanced i.v. fluids | Cumulative daily amount, unit | Once for each day of treatment with vancomycin and 2 days prior |
|  | Administration of i.v. 0.9% saline |  |  |
|  | Administration of i.v. glucose |  |  |
|  | Administration of any blood components except fibrinogen |  |  |
|  | Administration of i.v. albumin | Amount of administered albumin in grams |  |
| **VARIOUS KIDNEY FUNCTION PARAMETERES** | Creatinine clearance calculated by the Cockcroft–Gault equation | Each measured value (using serum creatinine values for calculation), unit, date and time | Each time novel measurements available during the treatment with vancomycin and 2 days prior |
|  | Creatinine clearance calculated by the Cockcroft–Gault equation. Corrected to body surface area |  |  |
|  | Creatinine clearance estimated by the Cockcroft– Gault equation with lean body weight |  |  |
|  | Glomerular filtration calculated by Chronic Kidney Disease Epidemiology Collaboration equation |  |  |
| **LABORATROY MEASUREMENTS** | Platelets count | Each measured value, unit, date and time | Each time measured during the treatment with vancomycin and 2 days prior |
|  | White blood cell count |  |  |
|  | Red blood cell count |  |  |
|  | Hemoglobin |  |  |
|  | Mean cellular volume |  |  |
|  | Serum creatinine |  |  |
|  | Hematocrit |  |  |
|  | Albumin |  |  |
|  | Uric acid |  |  |
|  | C-reactive protein |  |  |
|  | HCO3 |  |  |
|  | pH |  |  |
|  | Lactate |  |  |
|  | Procalcitonin |  |  |
|  | Sodium |  |  |
|  | Potassium |  |  |
|  | Isolation of G+ bacteria during the treatment with vancomycin or 2 days prior |  |  |

BW, body weight; IBW ideal bodyweight; ECMO, extracorporeal membrane oxygenation; ICU, intensive care unit; i.v., intravenous; p.o. oral.

**Table S2.** Prediction precision and bias shown for the 6 models that were selected by the genetic algorithm if they would be used alone. Mean PAPE, mean absolute percentage prediction error; mean PPE, mean percentage prediction error.

| **Name of the model** | **Mean PPE** | **Mean PAPE** |
| --- | --- | --- |
| **Kim et al.** | 14.35 | 25.82 |
| **Staatz et al.** | 36.39 | 41.06 |
| **Zhou et al.** | 4.66 | 26.00 |
| **Lin et al.** | 53.82 | 58.29 |
| **Usman et al.** | 5.07 | 53.41 |
| **Munir et al.** | -12.96 | 58.83 |

**Table S3.** The models final subsets selected by the genetic algorithm. The final MSTs was made using a group of models that had the lowest mean of the mean PAPEs of 10 CV sets by the end of the genetic algorithm (marked in bold). The precision of classification regression trees calculated with each subset, are shown in table.

| **Number of models in subset (M)** | **Mean of the mean PAPEs of the final subset of models in genetic algorithm** | **Precision of models predicted by the classification regression tree developed with final subsets of models**  **(mean PAPE (%))** | | **Models included in final subsets** |
| --- | --- | --- | --- | --- |
|  |  | **Training dataset** | **Validation dataset** |  |
| 4 | 23.4 | 23.0 | 29.1 | Kim et al.; Zhou et al.; Adane et al.; Deng et al. |
| 5 | 23.1 | 22.6 | 28.3 | Kim et al.; Staatz et al.; Zhou et al.; Munir et al.; Kovacevic et al. |
| **6** | **22.8** | **22.7** | **28.3** | **Kim et al.; Staatz et al.; Zhou et al.; Lin et al. (2021); Usman et al.; Munir et al.** |
| 7 | 23.2 | 22.7 | 28.4 | Kim et al.; Staatz et al.; Zhou et al.; Munir et al.; Buelga et al.; Lin et al.(2016); Wu et al. |
| 8 | 23.1 | 23.0 | 29.3 | Kim et al.; Zhou et al.; Usman et al.; Munir et al.; Alqahtani et al.(no-carcinoma); Buelga et al.; Deng et al.; Kovacevic et al. |

CV, cross validation; mean PAPE, mean absolute percentage prediction error.

**Table S4.** Predicting concentration-time data of the third measured concentration, which is blinded to the model/algorithm, in the assessment cohorts training and validation datasets in various settings: (1) a priori prediction using only the patient covariates; (2) prediction using patients covariates and the first plasma vancomycin concentrations; (3) prediction using patients covariates and the second plasma vancomycin concentrations and (4) prediction using patients covariates and both plasma vancomycin concentrations. Predictions with final MST and models used in final MST are presented.

|  |  | **Training dataset** | | | | **Validation dataset** | | | | |  |
| --- | --- | --- | --- | --- | --- | --- | --- | --- | --- | --- | --- |
|  | **Setting number** | **MST** | **Kim et al.** | **Zhou et al.** | **Staatz et al.** | | **MST** | **Kim et al.** | **Zhou et al.** | **Staatz et al.** | |
| **Mean PPE** | **1** | 14.3 | 22.2 | 12.6 | 41.7 | | 0.4 | 5.0 | 5.4 | 28.2 | |
|  | **2** | -2.4 | 18.3 | -4.8 | 23.4 | | -8.4 | 1.9 | -8.1 | 19.7 | |
|  | **3** | -4.5 | 9.2 | -6.8 | 6.2 | | -15.8 | -2.9 | -14.4 | 8.0 | |
|  | **4** | -3.0 | 13.1 | -6.0 | 12.3 | | -1.0 | -4.7 | 4.2 | 10.7 | |
| **Mean PAPE** | **1** | 29.6 | 30.5 | 32.5 | 44.2 | | 32.7 | 26.1 | 30.3 | 40.3 | |
|  | **2** | 21.9 | 25.6 | 20.1 | 28.3 | | 25.7 | 26.3 | 24.2 | 30.0 | |
|  | **3** | 22.0 | 19.2 | 21.7 | 19.3 | | 30.1 | 26.0 | 27.4 | 24.2 | |
|  | **4** | 17.0 | 20.2 | 16.3 | 17.6 | | 18.9 | 26.6 | 12.9 | 20.3 | |

Mean PAPE, mean absolute percentage prediction error; mean PPE, mean percentage prediction error; MST, model selection tool.
